# [Expression of Concern] Chrysophanol inhibits proliferation and induces apoptosis through NF-κB/cyclin D1 and NF-κB/Bcl-2 signaling cascade in breast cancer cell lines

**DOI:** 10.3892/mmr.2026.13966

**Published:** 2026-07-15

**Authors:** Li Ren, Zhouping Li, Chunmei Dai, Danyu Zhao, Yanjie Wang, Chunyu Ma, Chun Liu

Mol Med Rep 17: 4376–4382, 2018; DOI: 10.3892/mmr.2018.8443

Subsequently to the publication of the above article, an interested reader drew to the authors' attention that, concerning the western blot data shown in Figs. 4 and 5 on p. 4380, it was unexpected that the authors would have detected cleaved caspase-3 in the experiments performed using the MCF-7 cell line, since it has been shown that MCF-7 cells do not express caspase-3 (for example, see the paper by Reiner U. Jänicke and colleagues entitled “MCF-7 breast carcinoma cells do not express caspase-3.” Breast Cancer Research and Treatment, vol. 117.1 (2009); p. 219–221).

The authors have been contacted by the Editorial Office to offer an explanation for these unexpected findings in their study, and we are awaiting their response. Owing to the fact that the Editorial Office has been made aware of potential issues surrounding the scientific integrity of this paper, we are issuing an Expression of Concern to notify readers of these potential problems while the Editorial Office continues to investigate this matter further.

